# Management of Idiopathic Lower Extremity Ulcers With Chronic Venous Stasis Ulcer Therapy

**DOI:** 10.7759/cureus.90309

**Published:** 2025-08-17

**Authors:** Mishalle F Rashid, Samantha R Steiss, Arthur J Vayer

**Affiliations:** 1 Medicine, Edward Via College of Osteopathic Medicine, Blacksburg, USA; 2 Surgery and Wound Care, Sentara Northern Virginia Medical Center, Woodbridge, USA

**Keywords:** chronic venous ulcers, chronic wound care, compression therapy, idiopathic ulcers, leg ulcers, lower extremity wounds, skin ulcer treatment, ulcer management, venous stasis, wound healing

## Abstract

Idiopathic lower extremity ulcers are a common yet challenging clinical condition that generally requires multidisciplinary treatment. In this case report, we present the case of a 77-year-old male patient with idiopathic lower limb ulcers of undetermined cause. The patient also had a notable medical comorbidity of essential hypertension and a past surgical history of nephrectomy for renal carcinoma, yet no past medical history of proven venous disease. Despite extensive diagnostic investigation, utilizing both vascular evaluation and wound swab cultures, the precise etiology of his ulcerations could not be determined. Due to the chronicity and nonhealing nature of the wounds, the ulcers were managed with a combination of the standard treatment modalities commonly used for venous stasis ulcers. The patient was managed with a trial of compression therapy, ulcer debridement, and high-tech dressing, after which he had remarkable improvement. This case illustrates the potential benefit of establishing a standardized treatment modality for lower extremity venous ulcers with an undetermined etiology.

## Introduction

Chronic ulcers of the lower limb are a frequent cause of morbidity among the elderly population [1,2]. Although the vast majority of these ulcers result from the established etiologies of chronic venous insufficiency, arterial disease, diabetes, or infection, some ulcers remain idiopathic [3]. Typically, the etiology of a lower extremity ulcer determines its treatment; therefore, defining etiology is paramount to these cases. However, when preliminary testing fails to ascertain a definite etiology, physicians are left with the dilemma of choosing what they presume will be the most beneficial treatment regimen on a case-by-case basis [4]. Venous stasis ulcers account for approximately 80% of all lower extremity ulcers [5]. This case reports a patient with idiopathic lower limb ulcers successfully treated using conventional venous stasis ulcer treatment modalities. It also emphasizes the importance and value of considering this treatment approach for other lower extremity ulcerations of unknown origin [6]. Such non-healing ulcers carry significant implications for the quality of life of a patient in terms of long-term pain, limited mobility, and risk of secondary infection. Furthermore, chronic ulcers have a substantial economic impact on the health system through extended treatment duration, frequent hospital attendance, and the need for multidisciplinary care. The lack of established guidelines to treat idiopathic ulcers reflects the need for case-based evidence to inform practice. This paper aims to contribute to that emerging body of evidence by examining the effectiveness of standard venous ulcer treatments in resolving ulcers with an unclear etiology.

## Case presentation

A 77-year-old hypertensive male with a history of remote nephrectomy for renal cell carcinoma presented to the clinic with a two-week history of lower extremity edema and three insidious bilateral lower extremity ulcerations. The patient was non-diabetic, had no peripheral arterial disease, and had no venous insufficiency of known clinical significance. On examination, circumferential ulcers on both lower aspects of the extremities were observed with exposed subcutaneous tissue, serosanguineous discharge, and moderate necrotic debris. Wound #1, on the left medial lower leg (1.5 cm x 1.3 cm x 0.2 cm), was open with exposed subcutaneous fat, had little granulation tissue (1-33%), and extensive necrotic tissue (67-100%). Wound #2, on the left lateral lower leg (2.5 cm x 2.5 cm x 0.1 cm), was open, with exposed subcutaneous fat, but lacked granulation tissue and was mostly necrotic. Wound #3, the largest, on the right lower lateral leg (6.8 cm x 7 cm x 0.3 cm), contained no granulation tissue and had significant necrosis (67-100%) but no tunneling or undermining. Each wound contained moderate serosanguineous drainage, well-defined borders, and no atypical periwound findings. Peripheral pulses were equal and of palpable quality, and ankle-brachial indices were normal, excluding significant arterial insufficiency [7]. The exact attributes of the wound, including wound measurements, depth, type of tissue, and periwound tissue, were documented at baseline and evaluated at each follow-up appointment. Over the treatment period of four months, progressive reduction of wound size, reduction of necrosis, and increase of granulation tissue were noted. Healing milestones were achieved at approximately week 4 (necrosis resolution in smaller ulcers), week 6 (granulation tissue in all ulcers), and week 16 (almost complete healing).

**Figure 1:**
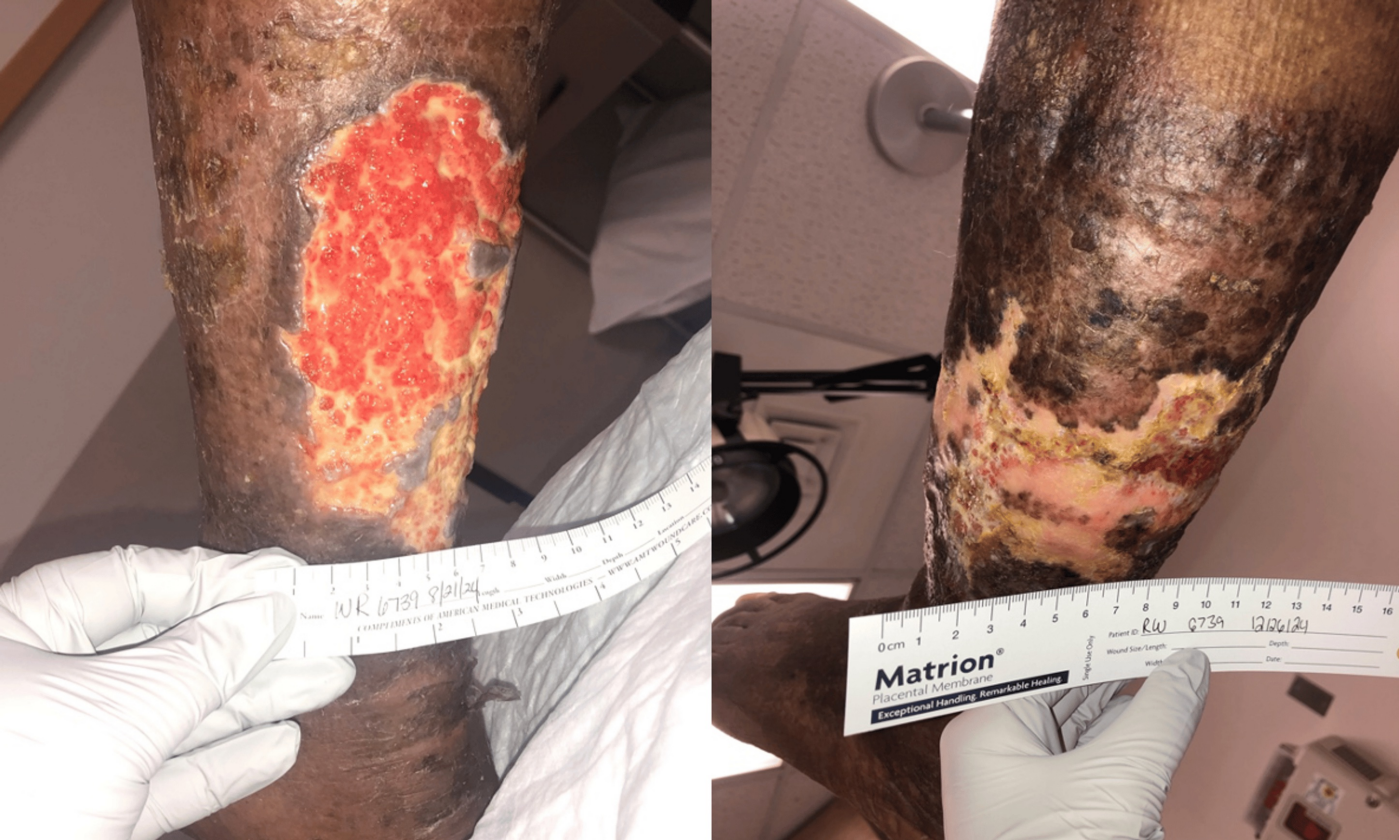
Lower extremity wound progression over a four-month period from 8/21/2024 (left) to 12/26/2024 (right)

Recent laboratory workup of inflammatory markers, autoimmune panels, and wound cultures (Erythrocyte sedimentation rate (ESR), C-reactive protein (CRP), myeloperoxidase antibody (MPO), anti-double stranded DNA (Anti-dsDNA), antinuclear antibody (ANA), proteinase 3 antibody (PR3), complement component 3 (C3), and complement component 4 (C4)) were within normal limits and could not contribute to the etiologic diagnosis of this patient. A venous duplex scan showed minimal valvular reflux, no deep vein thrombosis, or clinically significant venous obstruction [8]. Despite the lack of apparent venous pathology to be diagnosed, this patient's clinical situation and demographics allowed significant consideration of a subclinical chronic venous component. In the absence of diagnostic confirmation and with an urgent need for treatment to prevent further deterioration of the patient's condition, the decision was made to treat this patient’s ulcers as if they were of venous-stasis origin [9]. This treatment regimen included debridement, moisturized dressings, and compression therapy. As demonstrated in Figure 1, the lower extremity wounds showed remarkable improvement over a four-month period, with significant reduction in wound size, increased granulation tissue formation, and resolution of necrotic tissue from 8/21/2024 (left) to 12/26/2024 (right).

## Discussion

Lower limb ulcers are treated primarily based on their etiology. Unfortunately, there are instances when the etiology remains undetermined despite a thorough diagnostic workup [10]. This case reiterates that treating idiopathic ulcers as if they were of venous etiology may lead to satisfactory clinical improvement [10]. The most frequent etiology of lower limb ulcers is chronic venous insufficiency, and without definitive diagnostic features, treatment of the veins may be beneficial regardless [6].

Compression therapy remains the cornerstone treatment of venous ulcers and, as exemplified by this case, may be the cornerstone for treating all idiopathic lower extremity ulcerations [7]. Moreover, trials have demonstrated superior healing with compression versus standard wound management. The patient's tolerance to compression therapy was a critical step. It demonstrated the function of patient compliance and education as important factors in the beneficence of treatment. Serial debridement and high-technology dressing were also applied to aid in the success of the healing process [8].

A recent study also supports the need for urgent and precise diagnostic testing in chronic lower limb ulcer treatment, particularly in cases where common therapy fails. The research highlights the lack of response of atypical ulcers to standard management protocols and their imitative nature to other types of ulcers, requiring skin biopsy and multidisciplinary evaluation in cases of evasive etiology. In cases such as these, where characteristics of the ulcer are not typical and diagnostic tests are not conclusive, the use of therapeutic trial-like empiric compression therapy is still justified. The authors are quick to observe that all studies, including plethysmography, duplex ultrasound, MRI, and skin biopsy, must be interpreted with clinical presentation in mind to guide treatment and minimize the delay and cost of healthcare [11].

In addition, an exhaustive review of atypical ulcers demonstrated that prompt, specific treatment significantly improves patient outcomes. The research supports a paradigm whereby refractory ulcers, specifically, ulcers that fail standard care, are subjected to aggressive diagnostic workup with immunohistochemistry, microbiological examination, and systemic evaluation for autoimmune, neoplastic, or hematologic disease. The authors report that many chronic ulcers have complex or non-traditional etiologies and underscore the value of evidence-based, multidisciplinary management. This aligns with our case, where empiric venous-directed therapy succeeded without an identifiable cause, again validating the role of standardized and individualized treatment protocols, despite the lack of objective verification [12].

While this report did not make use of wound scoring tools, such as PUSH (Pressure Ulcer Scale for Healing), BWAT (Bates-Jensen Wound Assessment Tool), or TIME (Tissue, Infection, Moisture, and Edge), wound size, tissue composition, and the progression of healing were evaluated clinically and over time. Causation between the intervention and the healing cannot be substantiated, as other factors or spontaneous improvement might have played a role. Despite this limitation, the healing timeline observed was aligned with what is documented in the literature for the treatment of venous ulcers.

## Conclusions

This case shows that empiric treatment of lower extremity ulcers of unknown etiology can be followed by significant improvement through empiric treatment of venous stasis ulcers. Empiric therapy for venous insufficiency is a valid treatment option for idiopathic ulcers of the lower extremity. The patient's nonspecific clinical presentation, when taken in the absence of definitive venous stasis evidence, is worthy of trial-based treatment modalities in doubtful cases. The treatment strategy is designed to facilitate healing, reduce complications, and alter the quality of the patient's life when the outcome of the routine diagnostic tests is doubtful. Future studies must investigate this regimen in a larger population to determine the generalizability and transferability of the results in this specific case.
